# MicroRNA Expression in Pseudoexfoliation Syndrome with the Use of Next-Generation Sequencing

**DOI:** 10.3390/genes13040582

**Published:** 2022-03-25

**Authors:** Martyna Tomczyk-Socha, Julia Kręcicka, Marta Misiuk-Hojło, Anna Turno-Kręcicka

**Affiliations:** 1 Department of Ophthalmology, Wroclaw Medical University, Borowska 213, 50-556 Wrocław, Poland; marta.misiuk-hojlo@umw.edu.pl (M.M.-H.); anna.turno-krecicka@umw.edu.pl (A.T.-K.); 2 Department and Clinic of Ophthalmology, Wroclaw University Hospital, Borowska 213, 50-556 Wrocław, Poland; julia.krecicka@gmail.com

**Keywords:** microRNA, miR, NGS, pseudoexfoliation syndrome, lens capsule

## Abstract

Pseudoexfoliation syndrome (PEX) is a clinically important and biologically intriguing systemic disorder of the extracellular matrix. PEX etiopathogenesis was proved to be connected to multiple genes and other factors. However, the exact etiopathogenesis remains unknown. The aim of this study was to analyze miR expression in PEX using next-generation sequencing. An attempt was made to find the most commonly occurring miR in PEX, to evaluate miR that may have an essential role in the etiology of PEX syndrome. In addition, the correlation between the selected miRs’ expressions and age was investigated. Anterior lens capsules were obtained during cataract surgery. Next-generation sequencing was conducted on Illumina MiSeq. The average age was 68.2 years (with standard deviation +/− 6.92 years). Ten miRs with the highest level of expression represent approx. 95% of all readings. Four miRs with statistically significant differences in expression between groups have been distinguished: miR-671-3p, miR374a-5p, miR-1307-5p and miR-708-5p. The relationship between the most frequent miRs’ expressions and age has been evaluated and no correlation has been detected. In view of the above, it seems reasonable to examine the influence of miR on the biogenesis of PEX. Further studies on miR-671-3p, miR-374a-5p, miR-1307-5p and miR-708-5p expression in PEX are needed.

## 1. Introduction

Pseudoexfoliation syndrome (PEX) is a clinically important and biologically intriguing systemic disorder of the extracellular matrix. In this condition, which is connected to age, abnormal material accumulates in the anterior part of the eyeball, as well as in other organs. For this reason, PEX is considered a systemic disease. PEX rarely occurs before the age of 50, and its incidence increases with age.

In the last 15 years, we have observed a rapid increase in interest in the subject of molecular research in PEX. Hundreds of genetic, molecular, cellular and biochemical tests were performed, including scanning the entire genome, with the involvement of large cohorts. PEX etiopathogenesis was proved to be connected with multiple genes and other factors. However, the exact etiopathogenesis remains unknown.

MicroRNAs (miRs) are single-stranded, non-coding, endogenous regulatory particles. They consist of approximately 21–23 nucleotides. Their major role is the post-transcriptional regulation of multiple genes’ expression through complementary or partially complementary binding 3‘mRNA [1]. A single miR is able to control the expression of multiple destination genes; simultaneously, a single mRNA strand or destination gene may be modulated by multiple miRs [2]. It has been estimated that more than one-third of genes coding proteins in human cells are regulated by miRs, and that genes coding miRs represent 1–5% of all genes in humans and animals [3]. According to numerous scientific reports, miRs play a significant role in the course of multiple senescence [4]. This proves the meaningful role of miRs in the formation, development and progression of many diseases, including tumors and other diseases, in which new material is produced, as in PEX [5]. 

In 2018, the first article concerning miR expression in PEX was published [6]. Chatzikyriakidou et al. have identified three polymorphisms connected with PEX, which are protective considering the risk of occurrence of PEX [6]. Furthermore, two polymorphisms in the 3′-UTR region of genes connected with miRNA biogenesis were considered to be associated with PEX or secondary pseudoexfoliation glaucoma (PEXG).

Drewry et al. presented a differential analysis of miR expression in the aqueous humor of patients with primary open-angle glaucoma and PEXG [7]. Patients with cataracts were enrolled as a control group. Researchers have identified 298 miRs, among which they distinguished those whose expression significantly varied between groups. In the group with PEXG, five miRs were discovered with considerably different expression when compared with the control group [7]. 

The aim of this study was to analyze miR expression in PEX using next-generation sequencing. An attempt was made to find the most commonly occurring miR in PEX and to evaluate miRs that may have an essential role in the etiology of PEX syndrome, whose expression should be examined closely in subsequent studies. In addition, the correlation between the level of selected miRs’ expressions and age was investigated.

## 2. Materials and Methods

The following inclusion criteria to the group of patients with PEX syndrome were considered: age > 50 years, diagnosis of PEX and cataract, no current ophthalmological treatment, no intercurrent diseases, excluding hypertension. Exclusion criteria were: surgical procedures in study eye in the past, intercurrent ophthalmological conditions in the study eye (at present or in the past), glaucoma, diagnosed and treated systemic diseases (excluding hypertension), use of ophthalmological medication on the study eye 6 months prior to the cataract surgery, with the exception of hydrating preservative-free drops.

People with similar anthropometric features (similar age, sex, without any ophthalmological disease, with the exception of cataract, without any systemic disease, with the exception of hypertension) were enrolled as a control group.

Each patient underwent complete ophthalmological examination (anamnesis, autorefractometry, keratometry, visual acuity examination, tonometry, anterior segment slit lamp examination before and after dilatation of pupils with 1% Tropicamide and 10% Phenylephrine, fundus examination) and was asked to complete a questionnaire (containing questions about age, sex, race, smoking and other addictions, ophthalmological anamnesis concerning used medications, especially antiglaucoma drugs, general anamnesis regarding medications and dietary supplements, surgical procedures and injuries in the past).

A central part of the lens’ anterior capsule with a 5–6 mm diameter was collected during a cataract phacoemulsification procedure under peribulbar anesthesia. The collected piece was then secured in a test tube containing RNAlater™ (Thermo Fisher Scientific, Waltham, MA, USA) fluid.

### 2.1. RNA Isolation and Next-Generation Sequencing

The lens capsule was suspended in Invitrogen™ RNAlater™ Stabilization Solution (Thermo Fisher Scientific, Waltham, MA, USA). Next, the samples were cryopulverized in liquid nitrogen using Retsch CryoMill (Haan, Germany); mirVana™ miRNA Isolation Kit (Thermo Fisher Scientific) was then used to extract miRNA. The quality and sizes of miRNA were checked on Fragment Analyzer before (DNF-471-0500 RNA Kit (15NT)) and after (DNF-915-0500 Kit) cDNA construction (Agilent, Santa Clara, CA, USA). Small RNA-Seq Library Prep Kit was used for cDNA preparation according to the manufacturer’s protocol (Lexogen, Vienna, Austria). Briefly, 3′ and 5′ adapters were ligated to the miRNA termini and reverse transcribed to generate cDNA. The cDNA was amplified and index sequences were added. Samples were size-selected on a 6% polyacrylamide gel, purified, quantified and pooled for multiplexed sequencing. The libraries were checked for size and quality with Fragment Analyzer using high sensitivity NGS Fragment kit ((DNF-474-0500 Kit). Pooled libraries were loaded onto flow cells for cluster generation on-instrument and subsequent sequencing on an Illumina MiSeq instrument (Illumina, San Diego, CA, USA). Fifty sequencing cycles were performed. Fastq files were generated by MiSeq Reporter software. Subsequently, miRBase Sequence was used (March 2018) and Bowtie2 software was utilized for genomic alignment of miR sequences.

### 2.2. Statistical Analysis

In statistical analysis, the Trimmed Mean of M (TMM) method was used in order to standardize readings. Prior to the selection of the standardization method, miRs with minor numerousness in all samples were removed from the analysis. A minimal threshold of counts per million (CPM) in at least 5 samples was used as filter criteria. The threshold was defined as 3.7 CPM, which, in the smallest trial, conforms with approx. 13 appearances in raw data. Function cpm from edgeR library was used.

In order to find genes with various expressions, the edgeR panel was used. MiRs differing in expression levels were separated, with the level of significance determined below 0.1 (*p* < 0.1). Correlations between age and miRs with the highest expression and between miRs with various expressions among groups were analyzed. Kendall’s correlations were used.

This prospective study was conducted with the approval of Bioethics Committee of Wroclaw Medical University (KB 272/2017 from 26 April 2017). All patients enrolled in the study have signed written informed consent. All collected data have been anonymized.

## 3. Results

Ten patients were enrolled in the study (five with PEX formed the study group and the other five formed the control group). The average age was 68.2 years (with a standard deviation +/− 6.92 years). The basic demographic data of the examined groups are presented in Table 1. No statistical difference has been shown between groups in terms of age and sex.

Ten miRs have been identified as presenting in the highest levels in the lens’ capsules of the PEX group. The data in Table 2 feature mean readings from all samples in every group examined after normalization.

**Figure 1:**
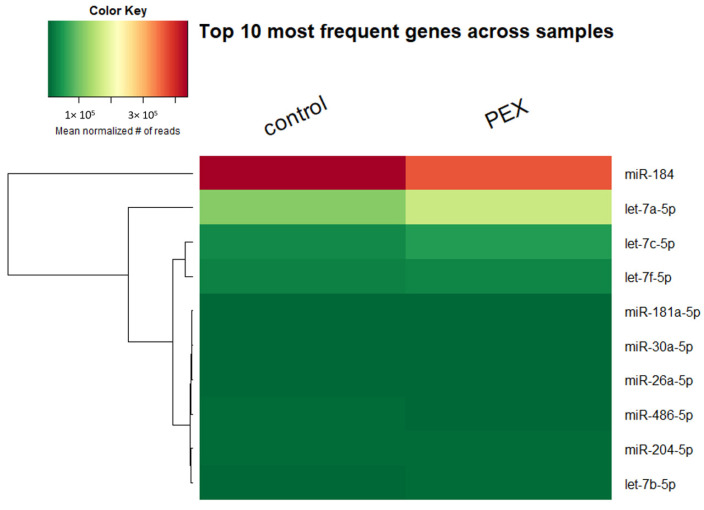
Heat map of most frequent miRs in PEX and control group.

The same 10 miRs occur in the highest level in the control group and the PEX group. These 10 miRs represent approx. 95%; the five most frequent represent 90% (Figure 1). The disclosed numerousnesses are mean numerousnesses normalized with the TMM method. No statistically significant difference has been shown between the expressions of the most commonly occurring miRs in either the PEX or control group (*p* < 0.05).

**Figure 2:**
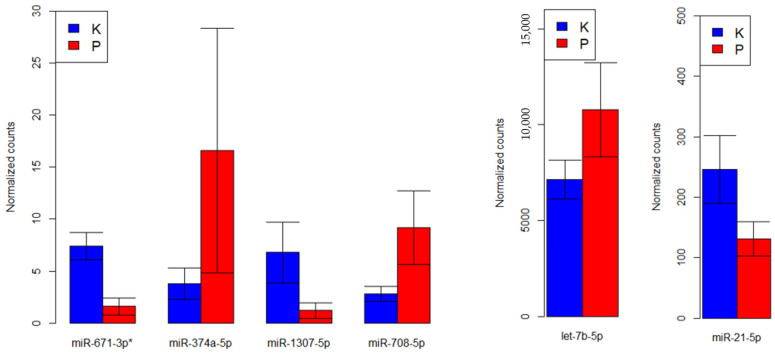
Expression of selected miRs with significant differences between PEX and control group. * miRs with *p* < 0.05 have been marked. At other times *p* < 0.1. (K—control group, P—PEX group).

**Figure 3:**
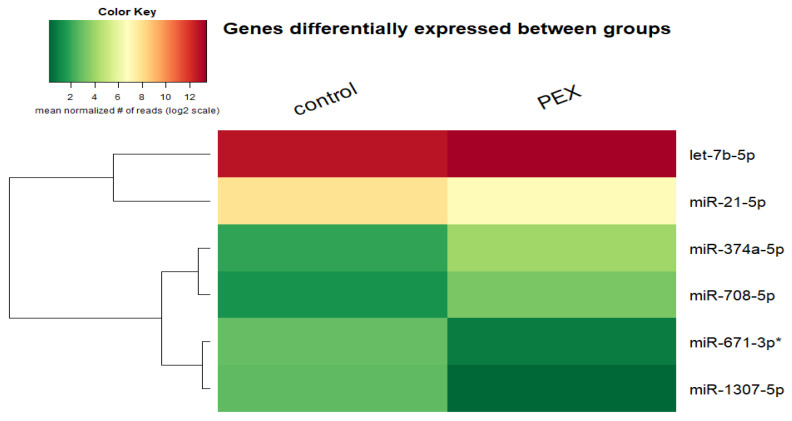
Heat map of miRs with statistically significant differences between PEX and control group. Values have been logarithmized with the aim of better visualization. * miRs with *p* < 0.05 have been marked.

In order to find genes with different expressions, the edgeR package (dispersion estimation, match of model (negative binominal) and the use of likelihood ratio test) was used. Statistically important differences in the expression of one miR in the PEX group in comparison to the control group have been shown with a significance level below 0.05, and six had a *p*-value below 0.1. miR-671-3p had significantly lower expression in PEX (*p* = 0.04). Moreover, the reduced expressions of miR-1307-5p (*p* = 0.08) and miR-21-5p (*p* = 0.09) and elevated expressions of miR-374a-5p (*p* = 0.07), miR-708-5p (*p* = 0.09) and let-7b-5p (*p* = 0.09) have been proved (Figure 2). Figure 3 demonstrates a heat map of miRs with statistically significant differences between the PEX and control group.

In order to identify miRs whose expression varies for groups examined using miSeq, many authors also analyze the logarithmized value fold change (logFC) in addition to the level of significance. This indicator does not analyze the level of significance, which is why it is connected with the *p*-value; logFC ≥ 1.5 or logFC ≤ 1.5 and *p*-value < 0.05 have been established as criteria. Four miRs (Table 3) with statistically significant differences in expression between groups have been distinguished: miR-671-3p, miR374a-5p, miR-1307-5p and miR-708-5p.

The relationship between the most frequent miRs’ expression and age was evaluated using Kendall’s tau coefficient. No correlation has been detected. Likewise, testing was conducted in the group of miRs with statistically significant differences in expression among groups. There was no correlation with age.

## 4. Discussion

For the first time, miRs have been sequenced in PEX using a next-generation sequencer. MiRs whose expression is different in PEX and the control group have been identified.

Despite molecular tests being thoroughly and widely performed in the last 20 years, the basic and most burning questions regarding PEX etiology still remain unresolved. Many scientists have released hundreds of research works trying to comprehend the etiopatogenesis of PEX. Researchers have distinguished many molecules, proteins, genes and impaired stress response pathways that have a significant effect in PEX. Still, it was impossible to determine the direct causative agent in PEX or to combine all discoveries into one process.

Using new technology such as next-generation sequencing (NGS), millions of short sequence readings can be obtained, such as miRs. This technique is applicable for profiling known miRs and describing new ones. NGS enables profiling the expression of miRs with unprecedented sensitivity and resolution (8). In comparison to the available miR microarray platforms, NGS systems are not limited by the preconceived number of features, probe construction, probe cross-hybridization or difficulties with matrix backdrop. Moreover, NGS systems directly calculate the number of transcripts found as a measure of the abundance of expressions, have a high potential for multiplexing, are not influenced by species, show high sensitivity for a low number of transcripts and have perfect reproducibility. Importantly, the data show that miR profiles of expression using miSeq and qPCR are very similar [8,9].

Despite the fact that PEX and cataracts are conditions connected to age, occur in elderly patients and are associated with the aging process, in this research work, there was no evident correlation between age and the expression of the most frequently appearing miRs and miRs with statistically significant differences in expression. However, it should be emphasized that the average age of patients was shifted toward higher values in relation to normal distribution. Furthermore, the number of patients was so small that conclusions concerning correlation with age should be drawn cautiously.

In previously published studies, Drewry (7) sequenced the level of miRs in pseudoexfoliative glaucoma, isolating material from aqueous humor. Significant statistical differences in the levels of miR-122-5p, miR-3144-3p, miR-320a, miR-320e and miR-630 expression have been confirmed. None of these miRNAs have been previously linked to glaucoma. In our study, the expression of those miRs has not been detected. However, it should be noted that the expression regarding the lens capsule, not aqueous humor, was examined. Additionally, the study group was composed of patients with pseudoexfoliation syndrome without glaucomatous damage. Therefore, the results may differ.

Studies that had been carried out on lens capsules in pseudoexfoliation syndrome have shown a statistically significant increase in miR-125b-5p levels [10]. In the current study, the level of miR-125b-5p expression has also been elevated, but this was not statistically relevant.

It has been proven that miRs play a role as regulative factors of multiple processes. Changes in their levels may regulate the concentration of proteins and other important particles responsible for the pathogenesis of diseases. Until now, the expression of individual miRs has been examined—those which were thought to have impact on certain disease pathogenesis. NGS enabled the new possibility of looking for important regulators. The current study also confirmed the thesis that the important role in disease regulation and genesis belongs not necessarily to those miRs whose concentration is highest in particular tissue, but also to those with lower expression, which are now possible to be found and selected thanks to NGS. Below, the meaningful influence of selected miRs on the pathogenesis of diseases is presented.

In previously published studies, miR-671-3p was identified as a potential biomarker in osteoarthritis involved in metabolic processes. It has been observed that, in breast cancer, the expression of miR-671-3p is increased, and this miR acts as a tumor suppressor affecting the signaling cascade Wnt. It was also stated that miR-671-3p regulates the proliferation, apoptosis, migration and invasion of non-small cell lung cancer cells by directly targeting FoXP2 (Forkhead box protein P2) [11]. In the literature, miR-671-3p has also been shown to regulate particles in the progression of glioblastoma by its effect on CKAP4 (cytoskeleton-associated protein 4) [12] and MMP-9 (matrix metallopeptidase 9) [13]. Data regarding the influence of miR-671-3p on tissues of the eye and ophthalmological diseases have not yet been published.

A decreased expression of miR-374a-5p has been observed in the plasma of patients affected by age-related macular degeneration [14]. Moreover, it has been stated that this miR can act as a potential modulator of inflammation in obesity. Thirty-seven protein genes have been distinguished as being part of pro-inflammatory pathways (IL-17A, chemokines, heat shock protein) and lipid metabolism [15]. miR-374a-5p is a potential prognostic marker in triple negative breast cancer. It aims directly at β-arrestin-1 (ARRB1) [16]. Furthermore, it has been concluded that the overexpression of miR-374a-5p decreases cell apoptosis by blocking the PTEN/PI3K pathway and participates in ischemic processes [17]. miR-374 facilitates proliferation and migration of transformed mesenchymal stem cells by regulation of the Wnt5a/β-catenin signaling pathway [18].

Among the survivors of Ebola virus disease, significantly increased expression of miR-1307-5p in retinal pigment epithelium (RPE) has been observed, which may be connected with repeat kinase, rich in leucine-2 [19]. miR-1307 may play a part in the development of resistance for chemotherapy in breast cancer by the modulation of apoptosis and targeting the Mdm4 protein [20]. This protein interacts with P53 through binding the domain localized in the N-terminal region of the MDM4 protein.

A negative correlation between LOXL1-AS1 and miR-708-5p expressions in breast cancer has been observed. An increase in LOXL1-AS1 facilitates the invasion of breast cancer and metastasis by blocking the expression and activity of miR-708-5p. LOXL1-AS1 is long, noncoding RNA coded on the opposite strand to LOXL1 (on the complementary strand) [21].

It has been proven that LOXL1-AS1 contains a promotor and has a strong relationship with high-risk alleles in PEX in the South African population. They constitute functional variants, which significantly modulate the activity of the promotor contained in this gene. LOXL1-AS1 expression is also relevantly changed in response to oxidative stress in the epithelial cells of the human lens and in response to cyclical mechanical stress in the endothelial cells of the human Schlemm’s canal. Researchers suggest that deregulation of LOXL1-AS1 expression may play the key role in PEX pathogenesis [22].

In the literature, the role of miR-708-5p has been identified as being a factor in pathogenesis and the progress of multiple tumours, particularly in transition from epithelial to mesenchymal form. The high expression of LOXL1-AS1 facilitates the occurrence of mesenchymal features of glioblastoma. LOXL1-AS1 modulates the proliferation of medulloblastoma’s cells by PI3K/AKT pathway activation. Overexpression of LOXL1-AS1 significantly lowers the expression of miR-142-5p, but increases the level of expression of PIK3CA in the neoplastic cells of the stomach [23].

Furthermore, it has been shown that, in gastric neoplastic cells, miR-708-5p expression is regulated by the long, noncoding particle MCM3AP-AS1, which regulates the proliferation and apoptosis of these cells [24].

A correlation between miR-708-5p and protein MAPK14 has been found [25]. Protein MAPK14 was examined earlier as a potential causative factor in PEX [26,27].

In addition, miR-708-5p promotes the apoptosis of synoviocytes, fibroblast-like cells, and eases rheumatoid arthritis by suppressing the Wnt3a/β-catenin pathway [28]. miR-708-5p also inhibits the creation of stem cell-like cells in lung cancer through the signaling Wnt/β-catenin pathway [29]. The Wnt signaling pathway consists of multiple cellular proteins, playing a part in embryogenesis and carcinogenesis, as well as in the physiological processes that take place in the normal cells of adult organisms [30].

Wnt3a/β-catenin and PI3K/AKT pathways are considered proinflammatory and profibrotic signaling pathways that are activated by reactive oxygen species, which play an important role in the pathogenesis of glaucoma [31]. Different miRs were described as the regulators of the expression of genes and proteins involved in the inflammatory and degenerative processes in glaucoma [31]. Specific miRNAs may be promising therapeutic targets for lowering or preventing oxidative stress injury.

In sum, the influence of miR-671-3p, miR-374a-3p, miR-708-5p on the Wnt/ β-catenin pathway, the influence of miR-374a-3p and miR-708-5p on PI3K and the significant role of miR-708-5p in LOXL1-AS1 have been demonstrated. What also seems to be interesting is the impact of miR-708-5p on MAPK14 and the impact of miR-1307-5p on P53 protein.

## 5. Conclusions

The available arguments suggest that PEX is an age-related, stress-induced microfibrylopathy, resulting from the excessive production and unarranged dispersion of pseudoexfoliation material. Despite multiple studies about PEX that have been conducted lately, we still do not know the etiology of PEX. The use of ophthalmological, anatomical and histological knowledge, as well as the latest technologies, still does not explain the etiopathogenesis of PEX.

An interesting and innovative aspect of this study was distinguishing four miRs with statistically significant differences in expression between groups—miR-671-3p, miR374a-5p, miR-1307-5p and miR-708-5p. These miRNAs may play a role in PEX pathways and act as biomarkers for disease pathogenesis or therapeutic targets.

## Figures and Tables

**Table 1:** Average age and gender distribution divided into groups.

Group	Number of Patients	Mean Age	Gender Distribution (Female:Male)
PEX	5	71.2	3:2
control	5	65.2	3:2
all patients	10	68.2	3:2

**Table 2:** Most frequently presented miRs in lens’ capsule. Data shown as the mean average based on readings from all samples in given group examined after normalization.

	Control Group	[%]	PEX Group	[%]
miR-184	440,360	61.9%	370,470	52.1%
let-7a-5p	118,218	16.6%	169,010	23.8%
let-7c-5p	39,434	5.5%	56,383	7.9%
let-7f-5p	29,825	4.2%	36,071	5.1%
miR-204-5p	12,044	1.7%	10,554	1.5%
let-7b-5p	7127	1.0%	10,797	1.5%
miR-486-5p	10,394	1.5%	5698	0.8%
miR-181a-5p	7123	1.0%	8266	1.2%
miR-30a-5p	6547	0.9%	7195	1.0%
miR-26a-5p	6814	1.0%	6001	0.8%

**Table 3:** logFC and significance levels of chosen miRs.

miR	logFC	*p* Value
miR-671-3p	−1.922801194	0.04157851
miR-374a-5p	2.051367227	0.07052728
miR-1307-5p	−1.964038782	0.07678925
miR-708-5p	1.658966927	0.08528887
miR-21-5p	−1.006061406	0.08898126
let-7b-5p	0.986972741	0.09325462

## Data Availability

Not applicable.
